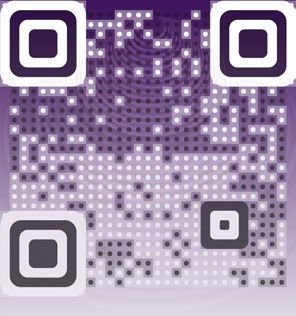# Continuing Professional Development—Medical Imaging

**DOI:** 10.1002/jmrs.886

**Published:** 2025-05-19

Maximise your continuing professional development (CPD) by reading the following selected article and answering the five questions. Please remember to self‐claim your CPD and retain your supporting evidence. Answers will be available via the QR code and published in JMRS—Volume 72, Issue 4, December 2025.

## Collaborative Use of a 3D Anatomy Platform to Motivate and Enhance Anatomy Learning in First‐Year Online Medical Sonography Students

Michelle Fenech, Nadia Mead, https://doi.org/10.1002/jmrs.848.

1. When learning and teaching anatomy—especially when identifying structures in medical imaging—which systematic approach is recommended?
  - Examine structures from superficial to deep
  - Identify structures from the midline outward to the periphery
  - First assess whether the image appears normal or abnormal
  - Begin with larger, more obvious structures and use them as reference points to locate smaller, detailed structures
2. According to this study, why do regular peer‐to‐peer interactions support learning?
  - They make learning more social, foster student connectedness to enhance the learning experience, promote growth, and support student autonomy
  - They help prevent students from becoming distracted
  - They allow students to collaboratively complete assessments
  - They help students identify who is knowledgeable and willing to share ideas
3. Which of the following is not one of the zones described in Vygotsky's Zone of Proximal Development theory?
  - The learner can do with guidance
  - The learner cannot do
  - The learner can do well
  - The learner can do unaided
4. Which theory of learning underpins transformative learning through the creation and recreation of knowledge?
  - Threshold concept of learning
  - Transformative learning theory
  - Metamorphosis pedagogy
  - Transition pedagogy
5. According to the study authors, why is supported online learning of anatomy advantageous for students?
  - It offers flexibility of pace, greater accessibility, and improved equitability when properly facilitated
  - It provides opportunities for tactile learning
  - It allows students to learn in isolation without distractions from others
  - It enables students to quickly Google answers to accelerate their learning

## Answers

Scan this QR code to find the answers.